# Electronic nicotine delivery system (ENDS) battery-related burns presenting to US emergency departments, 2016

**DOI:** 10.1186/s40621-018-0135-1

**Published:** 2018-03-05

**Authors:** Catherine G. Corey, Joanne T. Chang, Brian L. Rostron

**Affiliations:** 1 Center for Tobacco Products, U.S. Food and Drug Administration, 10903 New Hampshire Avenue, Silver Spring, MD 20993 USA; 2 Center for Tobacco Products, U.S. Food and Drug Administration, Document Control Center, Building 71, Room G335, 10903 New Hampshire Avenue, Silver Spring, MD 20993-0002 USA

## Abstract

**Background:**

Currently, an estimated 7.9 million US adults use electronic nicotine delivery systems (ENDS). Although published reports have identified fires and explosions related to use of ENDS since 2009, these reports do not provide national estimates of burn injuries associated with ENDS batteries in the US.

**Findings:**

We analyzed nationally representative data provided in the National Electronic Injury Surveillance System (NEISS) to estimate the number of US emergency department (ED) visits for burn injuries associated with ENDS batteries. We reviewed the case narrative field to gain additional insights into the circumstances of the burn injury. In 2016, 26 ENDS battery-related burn cases were captured by NEISS, which translates to a national estimate of 1007 (95%CI: 357–1657) injuries presenting in US EDs. Most of the burns were thermal burns (80.4%) and occurred to the upper leg/lower trunk (77.3%). Examination of the case narrative field indicated that at least 20 of the burn injuries occurred while ENDS batteries were in the user’s pocket.

**Conclusions:**

Our study provides valuable information for understanding the current burden of ENDS battery-related burn injuries treated in US EDs. The nature and circumstances of the injuries suggest these incidents were unintentional and would potentially be prevented through battery design requirements, battery testing standards and public education related to ENDS battery safety.

## Background

Electronic nicotine delivery systems (ENDS) also commonly referred to as “e-cigarettes” or “vape pens” are products that use a battery to heat a nicotine-containing liquid into an aerosol that the user then inhales (FDA 2018). In 2015, an estimated 3.5% (7.9 million) US adults were current users of ENDS products (Phillips et al. 2017). Under certain conditions, short circuits or changes to the battery chemistry can occur, which can cause ENDS batteries to overheat and to vent or explode, potentially causing scalds, flame or contact burns, chemical burns, or blast injuries to the ENDS user (Walsh et al. 2016; Brownson et al. 2016).

In an assessment of events reported to federal agencies, the media, and the published literature, Rudy and Durmowicz (2016) identified 34 burn injuries to ENDS users and five injuries to non-users in the US from 2009 through September 2015. The US Fire Association found that 195 explosion and fire events involving ENDS were reported in the US media from 2009 to 2016, resulting in 133 acute injuries, 38 of which were severe (McKenna 2017). Toy et al. (2017) analyzed data from a burn registry in southern California and determined that there were 25 patients with burn injuries involving ENDS at a burn center serving six counties from November 2015 to March 2017. Most burns (72%) occurred when the ENDS exploded in the user’s pants pocket. The estimate from Toy is consistent with a report of 15 patients with injuries from ENDS explosions treated at the University of Washington Medical Center in Seattle from October 2015 to June 2016 (Brownson et al. 2016). These published reports, although informative, do not provide comprehensive and current totals of burn events related to ENDS in the US as a whole. To address this gap in the research literature, we analyzed nationally representative data to estimate the number of US emergency department (ED) visits for burn injuries associated with ENDS batteries.

## Methods

We analyzed information provided in the National Electronic Injury Surveillance System (NEISS), maintained by the US Consumer Product Safety Commission (CPSC). For NEISS, injury data is gathered from approximately 100 US hospitals selected as a probability sample of the roughly 5000 hospitals with EDs to provide national estimates of the number of injuries presenting to EDs (US CPSC 2017a). Each selected hospital collects information for patients treated for an injury; information includes a description of the consumer product associated with the injury, patient demographics, diagnosis, disposition, affected body part, and a 142-character narrative of the incident. We analyzed the 2016 NEISS public use data file (US CPSC 2017b).

To identify burn injuries associated with the use of ENDS we first applied a text search to the NEISS case narrative field. We retrieved cases containing any of the following terms: “cig”, “vape”, “vapor”, “ENDS” or “electronic nicotine device”. Our query did not rely on NEISS product codes. The existing product codes were either: not applicable (e.g., electronic cigarettes under product code 1932 “other drug and medications” is used, according to the coding manual, “only for poisonings and chemical burns to children under 5 years of age”), were too broad (e.g. product codes 884 and 883 pertain to any batteries and any battery chargers, respectively), or were not intuitive (e.g., product code 1645 for clothing was applied to burn injuries associated with ENDS failures occurring while the device was in the user’s pocket). Next we restricted to burn-related incidents (diagnosis codes: 46, 47, 48, 49, and 51). Finally, two authors manually reviewed the remaining cases and excluded burns associated with products not in scope including cigarettes and humidifiers. Among the ENDS-related burn cases, we reviewed the narrative field to gain additional insights into the circumstances of the injury.

For this analysis, age groups were categorized as younger than 18 years, 18–24 years, 25–54 years, and 55 years or older. Affected body regions were grouped as upper leg/lower trunk, hand/lower arm, and other body parts (which consisted of the face and lower leg) as these were the most common injury sites. Burn type was reported as thermal, chemical or electrical. Text searches were conducted using the SAS “FIND” function (version 9.4; SAS Institute, Cary, NC). All data analyses were conducted in SAS using PROC SURVEY to account for the sample weights and complex sample design for hospitals. Counts, weighted proportions and 95% confidence intervals (CIs) were calculated. Because only anonymized, publicly-available data were used, the study was considered exempt from human subjects committee review.

## Results

In 2016, 26 ENDS battery-related burn cases were captured by NEISS, which translates to a national estimate of 1007 (95% CI: 357–1657) injuries presenting in US EDs. Nearly all injuries occurred among males (98.5%) and those aged 54 years or younger (98.5%), including 18.9% who were younger than 18 years (Table 1). The most common burn type was thermal burns (80.4%), while the remainder were chemical or electrical burns. Most injuries occurred to the upper leg/lower trunk (77.3%), followed by the hand or lower arm (19.7%), and other body parts (3.1%). Injuries resulted in hospitalization for more than one-quarter (27.6%) of ED patients. Our examination of the case narrative field indicated that at least 20 of the burn injuries occurred while ENDS batteries were in the user’s pocket.

**Table 1:** Electronic nicotine delivery system (ENDS) battery-related burns presenting to US emergency departments, NEISS 2016

Characteristic	Unweighted counts (n)	National estimate ^a^	National % (95%Cl)
Age (in years)			
Younger than 18	3	190	18.9 (12.2–25.6)
18–24	4	109	10.8 (0.0–24.8)
25–54	18	693	68.8 (58.7–78.9)
55 and older	1	15	1.5 (0.0–5.1)
Sex			
Male	25	992	98.5 (95.1–100.0)
Female	1	15	1.5 (0.0–4.9)
Burn Type			
Thermal burn	22	809	80.4 (53.2–100.0)
Chemical burn	3	134	13.3 (0.0–38.3)
Electric burn	1	64	6.3 (0.0–19.9)
Affected Body Part			
Upper leg/lower trunk	19	778	77.3 (60.4–94.2)
Hand/lower arm	5	198	19.7 (2.0–37.3)
Other body parts^b^	2	31	3.1 (0.0–7.3)
Disposition			
Treated and discharged	13	626	62.2 (28.9–95.5)
Hospitalized	12	278	27.6 (2.6–52.5)
Other^c^	1	103	10.3 (0.0–34.7)
Total	26	1007	100.0%

^a^National estimates were produced by applying statistical weights provided by US CSPC’s NEISS to the unweighted counts

^b^Other body parts pertain to face or lower leg

^c^Other disposition refers to a patient that left the ED without being seen

Note: CPSC considers a national estimate unstable and potentially unreliable when the weighted estimate is less than 1200

## Discussion

We analyzed nationally representative data to estimate the number of ED visits in the US for burn injuries associated with ENDS batteries and found that approximately 1000 injuries occurred in 2016. Most of the burns from ENDS were thermal burns and occurred to the upper leg/lower trunk, and more than one-quarter of ED patients were hospitalized. Our examination of the case narrative field suggested that ENDS-related burn injuries frequently occurred while the ENDS device or batteries were in the user’s pockets, suggesting that these incidents were unintentional. A battery coming into contact with metal objects, such as loose coins in pants pockets, can create an external short circuit which can cause the battery to overheat and to vent or explode (Walsh et al. 2016). These types of incidents can potentially be prevented by implementing battery design requirements and battery testing standards (Underwriters Laboratories 2011). Consumer education on safe practices for carrying and operating ENDS devices and batteries may also prevent some battery failures.

These estimates of burden do not account for consumers who sustained burns injuries from ENDS batteries but were not treated in EDs including those with less-severe ENDS-related burns as well as those with more severe burns who were treated at specialty burns centers in 2016. In the absence of a product code specific to ENDS (i.e., one that is not specific to child poisonings and chemical burns), we used broad terminology in our text search to capture all potential ENDS-related burn cases although incidents may have been omitted. A product code specific to ENDS could be informative for future surveillance activities. Despite these considerations, this analysis extends prior research by using a nationally representative data source to characterize the current burden and nature of ENDS battery-related burn injuries in the US.

## Abbreviations

CI: confidence interval
ED: emergency department
ENDS: electronic nicotine delivery system
NEISS: National Electronic Injury Surveillance System
US CPSC: United States Consumer Product Safety Commission
